# Susceptibility to Medium-Chain Fatty Acids Is Associated with Trisomy of Chromosome 7 in *Candida albicans*

**DOI:** 10.1128/mSphere.00402-19

**Published:** 2019-06-26

**Authors:** Qinxi Ma, Mihaela Ola, Elise Iracane, Geraldine Butler

**Affiliations:** a School of Biomolecular and Biomedical Science, Conway Institute, University College Dublin, Belfield, Dublin, Ireland; Carnegie Mellon University

**Keywords:** *Candida albicans*, aneuploidy, fatty acids

## Abstract

Fatty acids have known antifungal effects and are used in over-the-counter topical treatments. Screening of a collection of gene knockouts in Candida albicans revealed that one strain, carrying a deletion of the transcription factor *DAL81*, is very susceptible to the medium-chain fatty acid undecanoic acid. However, reintroducing *DAL81* does not restore resistance, and editing *DAL81* in a different background does not introduce sensitivity. Whole-genome sequencing revealed that the C. albicans *dal81Δ/Δ* strain has an extra copy of chromosomes 5 and 7. Reversion to resistance to undecanoic acid was induced by growing the sensitive strain in yeast extract-peptone-dextrose with 60 μg/ml undecanoic acid for up to 9 days. Nine isolates that regained some resistance to undecanoic acid lost one copy of chromosome 7. The copy number of chromosome 5 does not appear to affect resistance to fatty acids. Moreover, the sensitivity may be related to having two copies of haplotype B of chromosome 7. In addition, we find that C. albicans strain SN152, used to delete *DAL81* and many other genes, has undergone a major loss of heterozygosity event on chromosome 2 and a smaller one on chromosome 3.

**IMPORTANCE** Aneuploidy (changes in chromosome number) and loss of heterozygosity (LOH) occur frequently in the human-pathogenic yeast Candida albicans and are associated with adaptation to stress and to antifungal drugs. Aneuploidy and LOH can also be induced during laboratory manipulations, such as during genetic transformation. We find that C. albicans strain SN152, commonly used to generate gene deletions, has undergone a major LOH event on chromosome 2. One deletion strain generated in this background has acquired extra copies of chromosomes 5 and 7. We find that trisomy (three copies) of chromosome 7 is associated with sensitivity to fatty acids.

## Abstract (precis)

Aneuploidy (changes in chromosome number) and loss of heterozygosity (LOH) occur frequently in the human-pathogenic yeast Candida albicans and are associated with adaptation to stress and to antifungal drugs. Aneuploidy and LOH can also be induced during laboratory manipulations, such as during genetic transformation. We find that C. albicans strain SN152, commonly used to generate gene deletions, has undergone a major LOH event on chromosome 2. One deletion strain generated in this background has acquired extra copies of chromosomes 5 and 7. We find that trisomy (three copies) of chromosome 7 is associated with sensitivity to fatty acids.

## INTRODUCTION

Whole-chromosome or segmental aneuploidy occurs frequently in a variety of organisms, including humans, where it is often associated with developmental abnormalities and reduced fitness, most likely due to different gene dosage causing imbalances in protein stoichiometry (1). In the budding yeast Saccharomyces cerevisiae, changes in chromosome number are commonly associated with reduced proliferation, particularly delays in G_1_ phase in the cell cycle (1, 2). Pavelka et al. (3) showed that, compared to euploid controls, most aneuploid strains grow poorly in rich medium at 23°C. Changes in chromosome copy number contribute to changes in the transcriptome and proteome (2–4). However, some aneuploid strains have a significant growth advantage over euploid cells under some conditions, such as extreme temperature, pH, nutrient shortage, or the presence of chemotherapeutic or antifungal drugs such as rapamycin, bleomycin, thiolutin, or fluconazole (3).

Candida albicans is a diploid yeast and its genome is organized into eight pairs of chromosomes. Notably, the C. albicans karyotype is very unstable (5). Reversible loss or gain of a specific chromosome promotes survival under some conditions, e.g., exposure to antifungal agents or toxic sugar supplementation (5–7). Selmecki et al. (6) showed that ∼50% of strains resistant to fluconazole carried at least one aneuploid chromosome. Trisomy of chromosome R confers resistance to azoles, including fluconazole, ketoconazole, and miconazole (8). This phenotype can be reversed when the extra copy of chromosome R is lost by serially passaging on drug-free medium.

Aneuploidy is common in clinical isolates of C. albicans, especially in ones from deep-seated infections (9). In particular, formation of an isochromosome composed of two left arms of chromosome 5 (i5L) confers resistance to fluconazole (9). This is due to the increased copy number of *ERG11* and the transcription factor *TAC1*, both located on the left arm of chromosome 5. As a result, there is overexpression of the fluconazole target encoded by *ERG11* and of the fluconazole efflux pumps encoded by *CDR1* and *CDR2*, which are regulated by *TAC1*. In addition, chromosome 5L (chr5L) contains some genes encoding predicted efflux pumps, such as Orf19.4144, an ATP-binding cassette transporter, and Orf19.1942, a predicted multidrug resistance transporter. Transcription analysis showed that expression of most genes on chr5L were increased relative to expression of genes on chr5R in a strain carrying i(5L) (6, 9). Monosomy of chromosome 5 also results in elevated levels of chitin and reduced levels of 1,3-β-glucan, as well as diminished ergosterol levels, conferring resistance to caspofungin in some laboratory strains (10). Recently, Anderson et al. (11) found that in some backgrounds, trisomy of chromosome 4, resulting in elevated expression of two putative drug efflux pumps *CDR11* and *QDR1*, contributes to resistance to fluconazole. In addition, trisomy of chromosome 2 is associated with cross-adaptation to multiple drugs, including hydroxyurea and caspofungin (12).

We investigate here the role of aneuploidy in determining sensitivity to medium-chain fatty acids (MCFAs) in C. albicans. Fatty acids have long been known to have antimicrobial activities. For example, lauric acid, oleic acid, and linoleic acid inhibit growth of bacteria, including Staphylococcus aureus, Staphylococcus epidermidis, Pseudomonas aeruginosa, and Escherichia coli (13). Exogenous fatty acids inhibit fatty acid biosynthetic pathways in the parasite Plasmodium falciparum, which causes the most severe and deadly form of malaria responsible for 90% of malaria-related deaths in Africa (14). Morbidoni et al. showed in 2006 (44) that exposure to 2-hexadecynoic acid blocks both fatty acid synthesis and degradation in mycobacteria, indicating that fatty acids could also be a potential antituberculosis therapy.

The observation that fatty acids have antifungal activity dates back to the 1950s, when Prince (15) showed that the MCFA undecylenic acid (UDA [undec-10-enoic acid]) inhibited the growth of C. albicans. UDA is currently the only fatty acid used as a cost-effective antifungal, and it is an ingredient of many topical over-the-counter antifungal drugs used to treat oral thrush and denture stomatitis (16). Treatment with UDA inhibits germ tube formation, interferes with fatty acid biosynthesis, and disrupts pH in C. albicans (16–18).

We found that a C. albicans strain carrying a deletion of the transcription factor *DAL81* that was generated as part of a large-scale deletion project (19) is sensitive to low levels of MCFAs. Here, we show that the sensitivity of this strain is not caused by the deletion of *DAL81* but instead is associated with trisomy of chromosome 7.

## RESULTS

**FIG 1:**
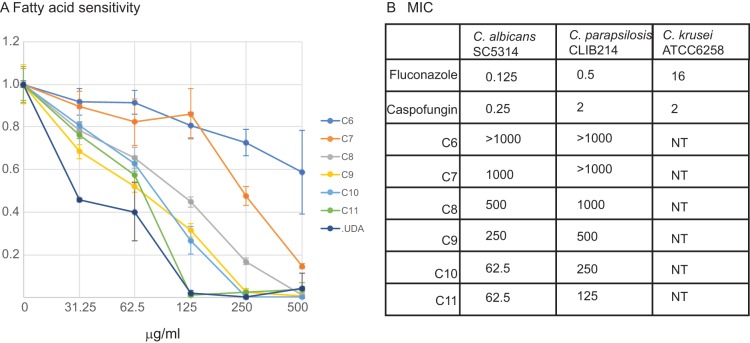
MCFAs inhibit growth of C. albicans. (A) C. albicans SC5314 was grown in YPD supplemented with free fatty acids (hexanoic acid [C_6_], heptanoic acid [C_7_], octanoic acid [C_8_], nonanoic acid [C_9_], decanoic acid [C_10_], undecanoic acid [C_11_], and undec-10-enoic acid [UDA]) at the indicated concentrations for 48 h. Growth in the absence of fatty acids was set to 1. The graphs show the averages and standard deviations of three independent measurements. (B) The MIC_50_ (μg/ml) was determined using the CLSI method. The effect of fatty acids was compared to fluconazole and caspofungin using the standard strains of C. parapsilosis and C. krusei, as recommended by CLSI.

UDA (undec-10-enoic acid or undecylenic acid), an MCFA with a single unsaturated bond, is used as a topical antifungal treatment for tinea pedis, a foot infection caused by dermatophyte fungi (20), and has been shown to inhibit growth of C. albicans in liquid culture and in biofilms (17, 18, 21, 22). We compared the anti-*Candida* effect of UDA to saturated MCFAs, with side chains that range from 6 to 11 carbons (Fig. 1). As shown previously (23), activity does improve with increasing chain length. However, MCFAs have poor antifungal activity compared to fluconazole or caspofungin, with MICs ranging from 62.5 to >1,000 μg/ml (Fig. 1). The effect of UDA is very similar to that of the saturated undecanoic acid (C_11_; Fig. 1A), and we used the latter in subsequent experiments.

**FIG 2:**
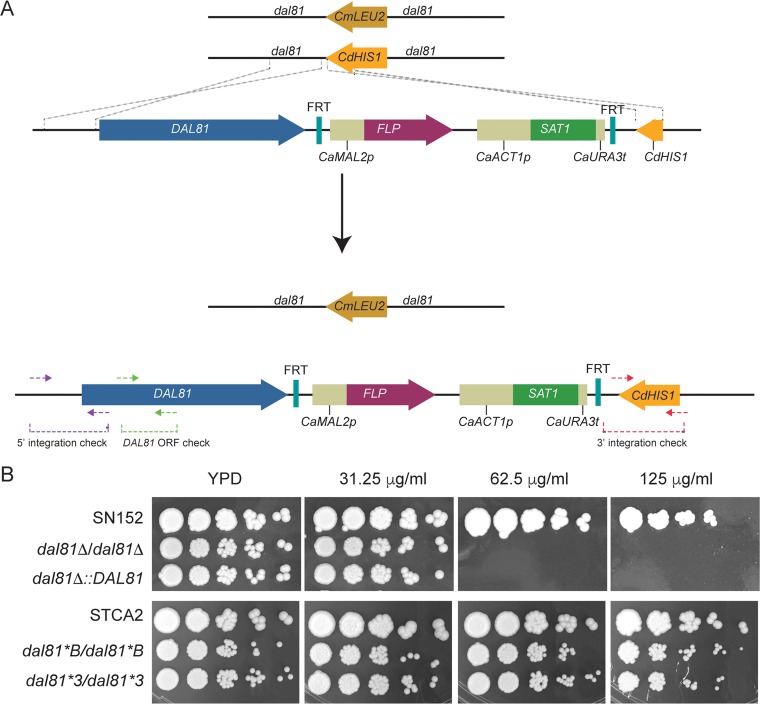
Sensitivity to fatty acids is not associated with deleting *DAL81.* (A) Reintroducing *DAL81* into the deletion strain. The *DAL81* alleles were originally deleted by replacing the open reading frames with *CmLEU2* and *CdHIS1* (19). To reintroduce the wild-type gene, the *SAT1* (nourseothricin resistance) cassette of plasmid pSFS2A (38) was flanked on one side with *DAL81* and on the other with part of *CdHIS1*. Homologous recombination results in integration of an intact copy of *DAL81* beside *CdHIS1*. The construct was confirmed by PCR using the indicated oligonucleotides and by genome sequencing. (B) C. albicans strains were grown overnight in YPD and increasing 1/5 dilutions were pinned on YPD and YPD containing undecanoic acid at the indicated concentrations. (Top panels) The *dal81* deletion strain from Homann et al. (19) is very sensitive to concentrations of ≥65 g/ml compared to the parental strain C. albicans SN152. Restoring one copy of *DAL81* did not affect the fatty acid sensitivity. (Bottom panels) Editing *DAL81* by introducing stop codons in a different genetic background (STCA2 [43]) did not result in fatty acid sensitivity. Two different edited versions of *DAL81* were tested (25).

To determine the pathways that control sensitivity to MCFA in C. albicans, we screened the collection of transcription factor knockouts constructed by Homann et al. (19) for sensitivity to undecanoic acid. We found that the C. albicans *dal81Δ/Δ* strain is sensitive to very low levels of undecanoic acid (31.25 μg/ml, Fig. 2). Dal81 is a transcription factor that regulates allantoin and GABA metabolism in S. cerevisiae (24) and arginine metabolism in *Candida* species (25). To determine whether fatty acid sensitivity is caused by deleting *DAL81*, we reintroduced a wild-type *DAL81* gene at one of the deleted alleles by homologous recombination (Fig. 2). The complemented strain remains sensitive to undecanoic acid at the same level as the *dal81Δ/Δ* strain, which indicates that the sensitivity is not caused by deleting *DAL81* (Fig. 2). In addition, a derivative of C. albicans SC5314 in which *DAL81* was edited by introducing two stop codons (25) did not result in sensitivity to undecanoic acid (Fig. 2).

### The *C. albicans dal81Δ/Δ* strain has three copies of chromosome 5 and chromosome 7.

The transcription factor knockouts described by Homann et al. (19) were generated in C. albicans SN152 (26). To determine the molecular mechanisms underlying the sensitivity of the C. albicans *dal81Δ/Δ* strain to undecanoic acid, we sequenced the genomes of the C. albicans SN152 and *dal81Δ/Δ* strains using Illumina and BGISeq platforms. All reads were compared to the C. albicans SC5314 reference assembly.

**FIG 3:**
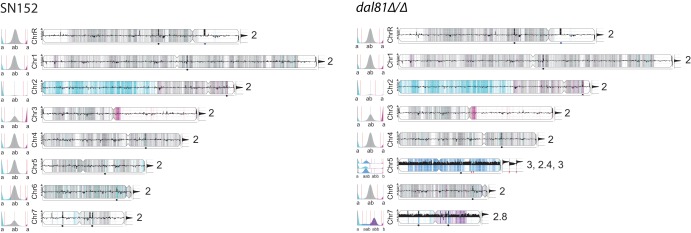
C. albicans *dal81Δ/Δ* is trisomic for chromosomes 5 and 7. The genomes of the C. albicans *dal81Δ/Δ* strain and the parental strain SN152 were determined using Illumina technology. The reads were mapped to the C. albicans S3514 reference genome, and SNPs were visualized using Y_MAP_ (27). Y_MAP_ distinguishes between the two haplotypes of the diploid chromosomes (A and B) and highlights copy number variations and aneuploidies using color. Heterozygous (AB) regions are shown in gray, homozygous AA regions are shown in cyan, and homozygous BB regions are shown in magenta. The black bars in the middle of the chromosomes, and the histograms at the right ends, indicate the copy numbers of each chromosome. For chromosome 5 in C. albicans, the copy number calculation varies from 2.4 to 3 because of a small dip in the calculation of the weighted average for a small part of the chromosome. The histograms on the left-hand ends show the proportion of each haplotype identified (marked as “a” or “b”).

We used Y_MAP_ (27) to visualize differences between the parental strain C. albicans SN152 and the *dal81Δ/Δ* derivative (Fig. 3). Y_MAP_ distinguishes between the haplotypes of each chromosome (A or B) using color. Heterozygous (AB) single nucleotide polymorphisms (SNPs) are shown as vertical gray bars in the background of each chromosome, and increasing shades of dark gray indicate regions with higher numbers of SNPs. Homozygous SNPs are displayed in cyan for haplotype A and in magenta for haplotype B. Aneuploidy is determined by the weighted average of the colors assigned to the individual SNPs. As shown in Fig. 3, there are two copies of all chromosomes in C. albicans SN152. However, a large region on chromosome 2 (colored in cyan) and a small region close to the centromere on chromosome 3 (colored in magenta) have undergone loss of heterozygosity (LOH). These LOH events happened at an early stage in the construction of C. albicans SN152 and not during passaging in our laboratory because they are also present in the C. albicans *dal81Δ/Δ* strain. In addition, the latter strain has gained an extra copy of haplotype A of chromosome 5 (shown in dark blue, the chromosome number is estimated to lie between 2.4 and 3) and an extra copy of haplotype B of chromosome 7 (shown in purple, the chromosome number is estimated as 2.8).

### Resistance to undecanoic acid is associated with chromosome loss.

To induce the restoration of resistance to undecanoic acid in the C. albicans *dal81Δ/Δ* strain, single colonies were randomly selected and inoculated in yeast extract-peptone-dextrose (YPD) supplemented with 60 μg/ml undecanoic acid. The cultures were diluted into fresh medium containing 60 μg/ml of undecanoic acid every 24 h, and single colonies were selected by spreading them on YPD agar plates containing 65 μg/ml undecanoic acid. Nine resistant colonies were selected for further investigation, originating from two different experiments after five to nine rounds of dilution. The genome of each isolate was sequenced, and the copy number of each chromosome was visualized using Y_MAP_ (27).

**FIG 4:**
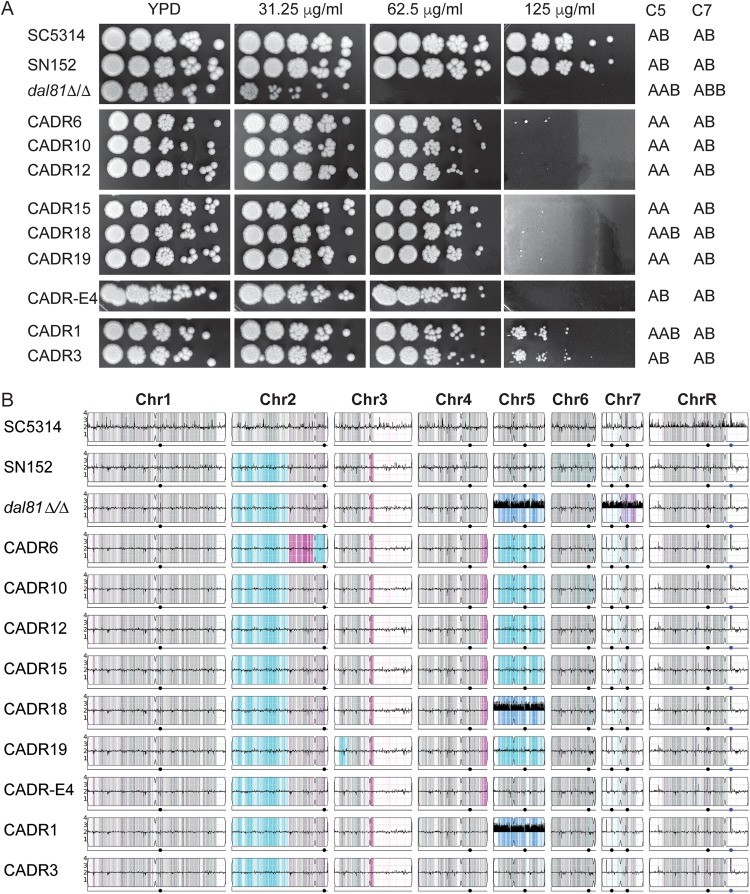
Resistance to undecanoic acid is associated with loss of chromosome 7 in the C. albicans *dal81Δ/Δ* strain. (A) Resistant isolates were selected from serial dilution in YPD broth supplemented with 60 μg/ml undecanoic acid. Sensitivity was determined by pinning on YPD and YPD containing undecanoic acid at the indicated concentrations, as described for Fig. 1. The chromosome haplotype and copy number are extracted from panel B. CADR1 and CADR3 were obtained from one replicate culture and the remaining strains from a different replicate. (B) Y_MAP_ analyses of the chromosome copy number and haplotype of the isolates shown in panel A. The LOH is shown in cyan and magenta, and trisomy of chromosome 5 is shown in dark blue and that of chromosome 7 is shown in purple. The chromosome copy number is also indicated by the bar in the center of the chromosome. Sequence reads for C. albicans SC5314 were obtained from the Sequence Read Archive (SRX747337).

The nine derivatives of the C. albicans *dal81Δ/Δ* strain from two separate experiments (CADR1, -3, -6, -10, -12, -15, -18, and -19 and CADR-E4) show increased resistance to fatty acids, and can grow in the presence of >60 μg/ml undecanoic acid (Fig. 4A). All of these isolates have lost one copy of haplotype B of chromosome 7 (Fig. 4B). Some have also undergone further changes. Five have lost haplotype B of chromosome 5 and retained two copies of haplotype A (CADR6, -10, -12, -15, and -19), two have lost one copy of haplotype A of chromosome 5 (CADR-E4 and CADR3), and two remain trisomic for chromosome 5 (CADR18 and -1). There are some additional LOH events on chromosomes 2, 3, and 4. However, the only event that is common to all isolates that have gained resistance is the loss of one copy of chromosome 7.

## DISCUSSION

Aneuploidy levels in C. albicans are high, and can be induced in the laboratory (28, 29). Arbour et al. (29) observed that each of the eight chromosomes of C. albicans can become duplicated in strains from different genetic backgrounds, such as CAI-4, RM1000, BWP17, or SN95, following a variety of treatments, including fluconazole treatment, Ura-blaster-mediated gene deletion, insertion of a PCR-amplified cassette, and MPA^R^ flipping. C. albicans CAI-4 is trisomic for chromosome 2, and more than half of transformants of this strain routinely lose the extra copy (30). Ahmad et al. (31) reported that some laboratory stocks of C. albicans SC5314 and its derivatives CAI4-2 and BWP17 differ in stability of chromosome R. No aneuploidies were reported in the auxotrophic strains C. albicans SN76, SN95, and SN152 (26), and they are generally recommended for genetic manipulation and mutational analysis (5, 29). We confirmed that C. albicans SN152 has no aneuploidies (Fig. 3). However, it has undergone a major LOH event in chromosome 2 and a smaller LOH near the centromere of chromosome 3. LOH can have major effects on phenotype (32). It is therefore important that the phenotypes of derivatives are compared directly to C. albicans SN152 and not to SC5314.

More importantly we found that the *dal81Δ/Δ* derived from C. albicans SN152 is trisomic for chromosomes 5 and 7 (Fig. 2 and 4). The changes in chromosome copy number in this strain may have occurred during the transformation when the deletion cassettes were introduced. Large-scale deletion collections provide wonderful resources for biological analyses (19, 25, 33–36). However, some unforeseen changes are likely to occur in some deletion strains during construction or during passaging and routine growth. When studying the effect of a specific gene deletion, it is therefore important to reintroduce the wild-type gene to determine whether the phenotype can be restored. Routinely testing for aneuploidy should be considered before planning subsequent experiments.

Fatty acid resistance was induced in the C. albicans *dal81Δ/Δ* strain by exposure to undecanoic acid for up to 9 days. We found that losing one copy of chromosome 7 restores some resistance in nine isolates. In all cases, the extra copy of haplotype B of chromosome 7 was lost (Fig. 4). It is therefore possible that fatty acid sensitivity is associated with having two B haplotypes, rather than trisomy. Alternatively, the B haplotype may be more prone to loss than the A haplotype. It is unlikely that chromosome 5 trisomy contributes to sensitivity because isolates with similar sensitivities have 2 (CADR3, -E4, -6, -10, -12, -15, and -19) or 3 (CADR1 and -18) copies of chromosome 5. However, the trisomy is clearly unstable, and chromosome loss occurs frequently.

How trisomy of chromosome 7 results in sensitivity to undecanoic acid is not clear. It may be related to the increased gene dosage, resulting in an imbalanced proteome, as shown in S. cerevisiae (2, 3, 37). Full resistance is not restored following loss of chromosome 7, suggesting that there may be other relevant variants in the C. albicans *dal81Δ/Δ* background. Further work is required to characterize the underlying mechanisms and to identify the specific genes on chromosome 7 that affect sensitivity to undecanoic acid in C. albicans.

## MATERIALS AND METHODS

### Strains and media.

All yeast strains (Table 1 ) were grown on YPD agar plates (Formedium, CCM0110) or in YPD broth medium (Formedium, CCM0210).

**TABLE 1:** *C. albicans* strains

Strain	Description or genotype	Source or reference
SC5314	Wild type	Type strain
SN152	*ura3/*::*imm434*::*URA3/ura3*::*imm434* *iro1*::*IRO1/iro1*::*imm434* *his1*::*hisG/his1*::*hisG leu2/leu2* *arg4/arg4*	19
*dal81Δ/Δ*	*ura3/*::*imm434*::*URA3/ura3*::*imm434* *iro1*::*IRO1/iro1*::*imm434* *his1*::*hisG/his1*::*hisG* *dal81*::*CdHIS1/dal81*::*CmLEU2*	19
*dal81Δ*::*DAL81*	*ura3/*::*imm434*::*URA3/ura3*::*imm434* *iro1*::*IRO1/iro1*::*imm434* *his1*::*hisG/his1*::*hisG* *dal81*::*DAL81/dal81*::*CmLEU2*	This study
CADR1, -3, -6, -10, -12, -15, -18, and -19 and CADR-E4	Fatty acid-resistant derivatives of *dal81Δ/Δ*	This study
STCA2	*ENO1/eno1*::*CAS9*	43
dal81*/*_B	*ENO1/eno1*::*CAS9 RP10*::*SAT1* dal81*/dal81*	25
dal81*/*_3	*ENO1/eno1*::*CAS9 RP10*::*SAT1* dal81*/dal81*	25

A wild-type copy of *DAL81* was reintroduced into the *DAL81* deletion strain of C. albicans (19) using the approach shown in Fig. 2. A fragment of the C. dubliniensis *HIS1* gene which was originally used to delete *DAL81* was cloned into plasmid pSFS2A (38) between SacII and SacI sites to generate the plasmid pSFS2A-CdHIS. *CaDAL81*, including ∼400 bp upstream, was amplified with the primers CaDAL81-KpnI-Fw and CaDAL81-BamHI-Rv (Table 2 ) and cloned into pSFS2A-CdHIS between the KpnI and BamHI sites. The resulting plasmid was digested with AgeI (which cuts inside the promoter region of *DAL81*) and SacI (which cuts at the 3′ end of the cloned CdHIS1 insert). The DNA fragment was transformed in the C. albicans *dal81Δ/Δ* strain using the lithium acetate chemical transformation method (39). Transformants were selected on YPD agar containing 200 μg/ml nourseothricin and incubated for 72 h at 30°C. Integration of *DAL81* was verified by PCR using primer CaDAL81-comp5V-Fw/Rv at the 5′ end and primer CdHIS1-comp3V-Fw/Rv at the 3′ end and was further confirmed by genome sequencing.

**TABLE 2:** Primers

Primer	Sequence (5′–3′)^*a*^
CaDAL81-KpnI-Fw	GAAggtaccGAGAGAGCGGCAATGAAATC
CaDAL81-BamHI-Rv	GATCggatccTCATTTGGTTAGTTCGGGTG
CaDAL81-comp5V-Fw	AGTGGTTGCCGTTTTTTTTC
CaDAL81-comp5V-Rv	TGGGTAGTGGGGATATTTGG
CdHIS1-comp3V-Fw	GATCCACTAGTTCTAGAGCG
CdHIS1-comp3V-Rv	GGGTGTTGCCGACGCCATTG

a Restriction sites are indicated in lowercase letters.

### Sensitivity assays.

For sensitivity assays, hexanoic acid (C_6_), heptanoic acid (C_7_), octanoic acid (C_8_), nonanoic acid (C_9_), decanoic acid (C_10_), undecanoic acid (C_11_), and undec-10-enoic acid (UDA; Sigma) were freshly prepared in methanol and diluted in 96-well plates to yield final concentrations of 1,000, 500, 250, 125, 62.5, and 31.25 μg/ml. Next, 100 μl of YPD with C. albicans cells at an *A*_600_ of 1 was added to each well. The plates were incubated at 30°C for 48 h, and the *A*_600_ was determined using a Synergy HT plate reader. Each sample was tested in triplicate. The growth without drug was set to 1.

The MICs that resulted in 50% inhibition of growth (MIC_50_) were determined using a standard Clinical and Laboratory Standards Institute (CLSI) assay method according to document M27-A3 (40). Inocula were prepared from a 24-h culture on Sabouraud dextrose agar plate, adjusted to the same absorbance as the 0.5 McFarland standard (CLSI, M27-S3) at 530 nm, and diluted in RPMI 1640 (Sigma, R1383) supplemented with 0.02% glucose (Sigma, G8720) to the desired concentration of 1 × 10^3^ to 5 × 10^3^ CFU/ml. Next, 100 μl of fatty acid was mixed with 100 μl of inoculum in a U-bottom 96-well plate, followed by incubation at 35°C. Plates were scored at 24 and 48 h as described in the CLSI document. The MIC_50_ was defined as the lowest concentration of drug that inhibits 50% growth relative to the growth in the drug-free control well after 48 h. Commercial antifungal drugs fluconazole (Sigma, F8929) and caspofungin (Sigma, SML0425) were tested against Candida parapsilosis CLIB214 and Candida krusei (ATCC 6258), as recommended by the CLSI to confirm the accuracy of the assays.

For drop test assays, yeast strains were grown overnight in 5 ml of YPD broth medium at 30°C with shaking at 200 rpm. Overnight cultures were collected by centrifugation at 13,300 rpm at room temperature for 1 min. Cells were washed twice with 1 ml of PBS and resuspended in 1 ml of PBS. Each cell suspension was adjusted to an *A*_600_ of 0.0625 in 1 ml of PBS and separated into aliquots at 200 μl per well of a 96-well plate. Strains were then serially diluted 1/5 in 200 μl of PBS to reach a final *A*_600_ of 0.0001. Then, 3 μl of each dilution was transferred onto solid agar plates containing different concentrations of undecanoic acid (31.25, 62.5, and 125 μg/ml) using a 48- or 96-pin replicator. Plates were incubated at 30°C and imaged after 72 h.

### Induction of resistance to undecanoic acid.

A single colony of the C. albicans *dal81Δ/Δ* strain (19) from a fresh culture on YPD agar was inoculated in triplicate in 5 ml of YPD medium and grown overnight at 30°C with shaking at 200 rpm. The experiment was repeated twice. The overnight cultures were inoculated to an *A*_600_ of 0.1 in 5 ml of YPD broth medium supplemented with 60 μg/ml undecanoic acid. Every 24 h, the culture was diluted to an *A*_600_ of 0.1 in 5 ml of YPD containing 60 μg/ml undecanoic acid. Every day, 100 μl of culture was spread onto YPD agar plates containing 65 μg/ml undecanoic acid in order to select resistant colonies. From experiment 1, we selected CADR6 (day 4), CADR10 and -12 (day 5), CADR15 (day 6), and CADR18 and -19 (day 8). CADR-E4 was also selected after day 4 in experiment 1, but the exact day was not recorded. CADR1 and CADR3 were selected after 4 and 5 days of successive dilutions, respectively, in experiment 2.

### Whole-genome sequencing and data analysis.

Genomic DNA was extracted from C. albicans SN152, *dal81Δ/Δ*, and *dal81Δ*::*DAL81* strains and 9 fatty acid-resistant revertants derived from the C. albicans *dal81Δ/Δ* strain (Table 1) using a QIAamp DNA minikit (Qiagen, Germany) following a customized protocol with minor changes. Whole-genome sequencing of the C. albicans SN152 and C. albicans *dal81Δ/Δ* strains was performed on an Illumina HiSeq-4000 sequencing platform by Beijing Genomics Institute (BGI). Approximately 5.7 million paired-end reads (150 bases) were obtained per sample. The remainder of the strains were sequenced by BGI using BGISeq500, with 100 base paired-end reads, yielding approximately 9 million reads per strain. Reads from C. albicans SC5314 were downloaded from the Sequence Read Archive (SRX747337), 100 base paired end, with 2 million reads. All reads were trimmed using Skewer version 0.1.120 with the following parameters: –m pe (paired-end mode), –l 75 (minimum read length allowed after trimming), –q 15 (trim 3′ end of read until a quality of 15 or higher is reached), and –Q 15 (lowest mean quality allowed for a read before trimming) (41). Trimmed reads were mapped to the C. albicans SC5314 reference genome using the “mem” algorithm from BWA with the parameters –k 75, –M, and –R (42) to generate BAM files. Chromosome copy number and loss of heterozygosity (LOH) was visualized by mapping the BAM files to the C. albicans SC5314 reference genome using Y_MAP_ (27).

### Data availability.

All sequencing reads were submitted to SRA (BioProject PRJNA546118).
