# Correction for Ma et al., “Susceptibility to Medium-Chain Fatty Acids Is Associated with Trisomy of Chromosome 7 in Candida albicans”

**DOI:** 10.1128/mSphere.00564-19

**Published:** 2019-08-14

**Authors:** Qinxi Ma, Mihaela Ola, Elise Iracane, Geraldine Butler

**Affiliations:** a School of Biomolecular and Biomedical Science, Conway Institute, University College Dublin, Belfield, Dublin, Ireland

## AUTHOR CORRECTION

Volume 4, no. 3, e00402-19, 2019, https://doi.org/10.1128/mSphere.00402-19. Following the publication of this paper, it came to our attention that we inadvertently failed to refer to some earlier work in the field. We described two loss-of-heterozygosity (LOH) events in the parental strain used in our experiments, Candida albicans SN152. However, we missed some earlier work that showed that these LOH events are present in a parental strain, C. albicans RM1000, and a related isolate, C. albicans SN76.

The following sentence, which includes a new reference citation, should be added as the 3rd-to-last sentence of the 1st paragraph of the Discussion section: “Abbey et al. (45) previously showed that the LOH events that we describe in C. albicans SN152 occurred in a parent of this isolate, C. albicans RM1000, and are also present in the related strain C. albicans SN76.”

The new reference is as follows:

45. Abbey D, Hickman M, Gresham D, Berman J. 2011. High-resolution SNP/CGH microarrays reveal the accumulation of loss of heterozygosity in commonly used Candida albicans strains. G3 (Bethesda) 1:523-530. https://doi.org/10.1534/g3.111.000885.

These changes have been made in the article reposted on 14 August 2019.

